# Seroprevalence of contagious bovine pleuropneumonia (CBPP) in cattle from Karamoja region, North-eastern Uganda

**DOI:** 10.1186/s12917-024-03938-8

**Published:** 2024-03-09

**Authors:** Robert Tweyongyere, Joseph Nkamwesiga, Patrick Etiang, Israel Mugezi, Henry Wamala, Auther Tamale Wasswa, Maureen Kamusiime, Solomon Ainebyoona, Harriet Abizera, Frank Norbert Mwiine, Dennis Muhanguzi

**Affiliations:** 1 College of Veterinary Medicine Animal Resources and Biosecurity, Makerere University, P.O.Box 7062, Kampala, Uganda; 2 Faculty of Agriculture and Animal Sciences, Busitema University, P.O.Box 236, Tororo, Uganda; 3 Department of Animal Health, Ministry of Agriculture Animal Industry and Fisheries, P.O.Box 513, Entebbe, Uganda; 4 Mercy Corps Uganda, Clock Tower, P.O.Box 32021, Kampala, Uganda; 5 Department of Production, Trade and Tourism Planning, National Planning Authority, P.O.Box 21434, Kampala, Uganda

**Keywords:** Karamoja region, Uganda, Seroprevalence, CBPP, cELISA, Cattle

## Abstract

**Background:**

Contagious bovine pleuropneumonia [CBPP] is a transboundary animal disease of cattle caused by *Mycoplasma mycoides subsp. mycoides* [Mmm]. CBPP causes severe economic losses to livestock producers in sub-Saharan Africa mainly due to high mortality, morbidity, reduction in productivity as well as livestock trade restrictions. This study aimed at determining seroprevalence of Mmm in cattle from Karamoja region, north-eastern Uganda; data that are required to design and implement risk based CBPP control program.

**Methods:**

We randomly collected blood samples from 2,300 cattle spread across Karamoja region. Serum was extracted and screened for antibodies against *Mycoplasma mycoides subsp. mycoides* [Mmm] using the competitive enzyme linked immunosorbent assay [cELISA].

**Results:**

A quarter [25.4%; 95% CI: 23.7–27.3] of the screened cattle [*n* = 2,300] were sero-positive for Mmm. Amudat and Kaabong districts recorded the lowest [12.3%] and highest [30.7%] Mmm seroprevalence respectively. Increasing age, overnight stay in cattle kraals and location [certain districts, villages, herds and sub counties] of the cattle herds, the factors that promote animal commingling, were the most significant risk factors of seroconversion with Mmm.

**Conclusion:**

Results from this study indicated a higher seroprevalence of Mmm in Karamoja region cattle herds. This could be due to the increased frequency of CBPP outbreaks in recent years. To be effective, CBPP vaccination programs should target high risk herds along the international borders and other hotspot areas [e.g., parishes or sub counties] where cattle commingling is high.

## Background

Contagious bovine pleuropneumonia is a transboundary animal disease of cattle caused by *Mycoplasma mycoides subsp. mycoides* (Mmm); a bacterium that has tropism for cattle respiratory system. Mmm is transmitted to susceptible cattle through inhalation of droplets from infected cattle [1]. CBPP is often acute or subacute; affected animals dying from pulmonary involvement that terminates into severe pneumonia and serofibrinous pleurisy. CBPP transmission is by direct contact from infected cattle [the only known reservoirs of infection]. Thus outbreaks are precipitated by animal movements that often occur during dry seasons when animals are stressed due to poor nutrition and exhaustion from trekking in search of pastures and water. CBPP is endemic in the most parts of Sub-Saharan Africa (SSA) including Uganda [2]. CBPP is thought to have been introduced into Uganda from South Sudan in 1956 when the first major outbreak occurred in Karamoja region [3]. Surveillance of CBPP outbreaks by the Ministry of Agriculture started this very year. This disease has since spread to the rest of the country. The factors that have driven CBPP spread from its initial Karamoja focus to the rest of the country have been cited as limited disease surveillance, minimal vaccination efforts and uncontrolled animal movements [3].

Aggregation of CBPP national passive surveillance data archived at the National Animal Disease Diagnostics and Epidemiology Centre [NADDEC], Ministry of Agriculture, Animal Industry and Fisheries [MAAIF] to determine spatial and temporal distribution of CBPP in Uganda (1956–2011) revealed that CBPP has been endemic in Uganda since 1956. Whereas between 1956 and 1974 reports of CBPP cases had been confined to the Karamoja region, the disease spread to other regions of Uganda and even to other East African countries due to uncontrolled cattle movements. There was a sharp increase [up to 21.9%] in the Mmm seroprevalence by CFT testing in Karamoja region between 1991 and 2001 indicating CBPP endemicity over time on sampling of Mmm-infected herds by the MAAIF surveillance teams [3].

The published CBPP seroprevalence estimates are over 10 years old and therefore not ideal for planning risk-based CBPP control programs. However, recent participatory epidemiology studies have identified that CBPP is one of the most important constraints to livestock health and production in Karamoja region [4, 5]; warranting estimation of the current seroprevalence of Mmm to justify risk-based CBPP control and surveillance programs. The economic cost of CBPP is estimated to be ∼ 44.8 million Euros in 12 endemic SSA countries [6]. The cost in each of the endemic countries varies widely. However, if CBPP control programs were instituted in each of the endemic countries, and Uganda in particular, the financial returns on investment would invariably be positive with benefit-cost analyses indicating a benefit-cost ratio approaching 2 [6]. To this end, we screened 38 cattle herds [*n* = 2,300] distributed in three districts [Amudat, Kaabong and Karenga] and determined the seroprevalence of CBPP in Karamoja region; findings we discuss herein regarding their usefulness in the design and implementation of risk-based CBPP control and surveillance programs.

## Methods

### Study area

**Fig. 1:**
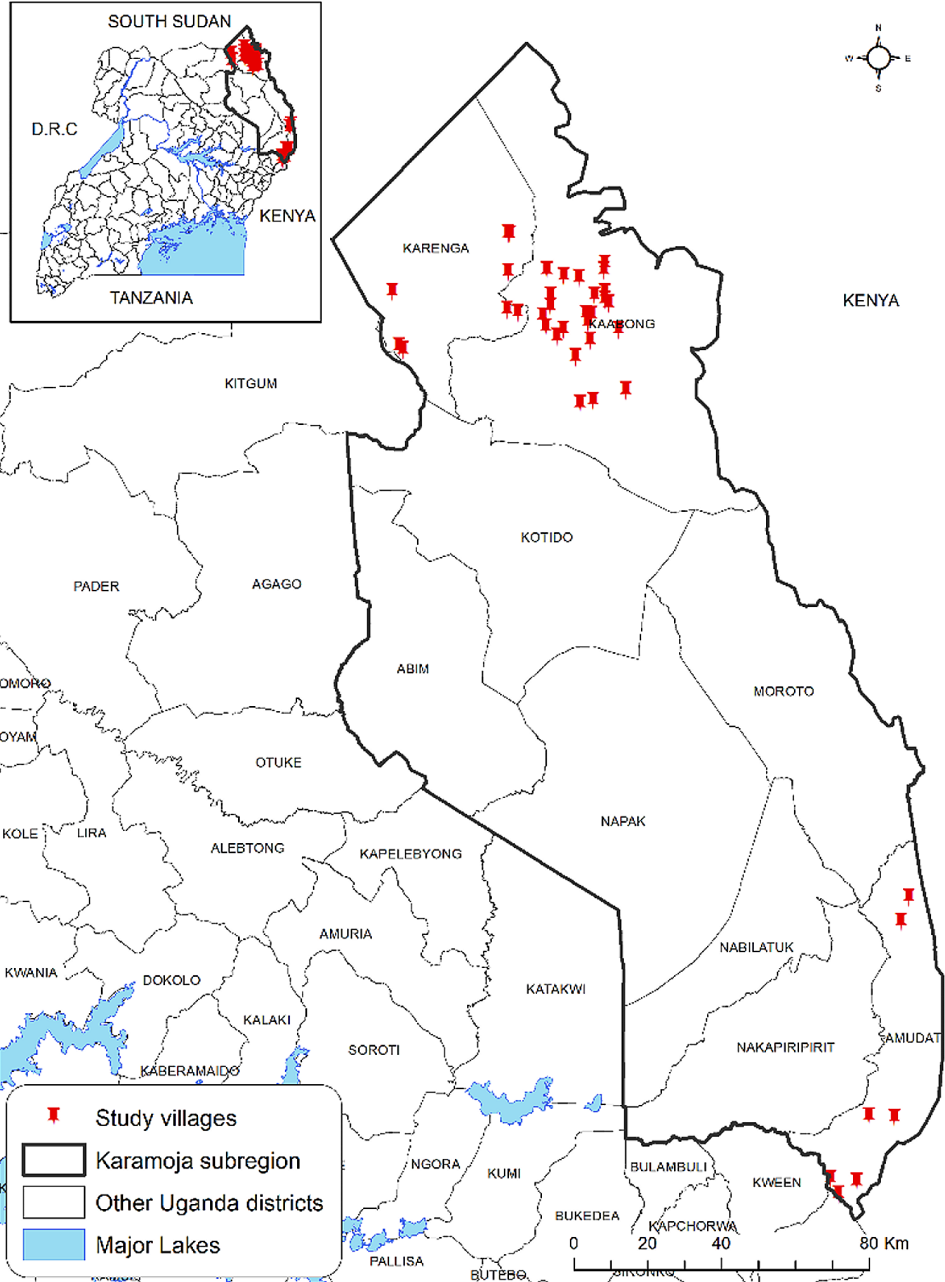
Study area: Red pins represent villages where biological samples were collected. This map was generated by the authors in ArcMap 10.7 software using open-source shape files https://data.humdata.org/dataset/uganda-administrative-boundaries-admin-1-admin-3

Karamoja region is a semi-arid region located in the north-eastern part of Uganda. Karamoja region is made up of nine administrative blocks [districts] namely, Kaabong, Karenga, Abim, Kotido, Moroto, Nabilatuk, Napak, Nakapiripiriti, and Amudat. This study was implemented in Amudat, Kaabong and Karenga districts [Fig. 1]. These districts were randomly selected from nine Karamoja region districts.

The region is characterized by a unimodal rainfall pattern with poor distribution and reliability. On average it receives 745 mm of rainfall per year (varying widely from 600 mm in the north to 1000 mm in the southern and western parts). The rainy season spans from April to September with scanty rains in June, a main peak in July/August and a minor peak in May, followed by an intense dry season typically with strong winds from October to April, with January as the driest month. Daily temperatures average 30–35^0^ C [7, 8]. The vegetation is Savannah dominated by seasonal grasses, thorny plants, and occasional small trees, with thickets and patches of gallery found along seasonal rivers [7].

### Study population

This study was carried out in thirty-eight of the 628 cattle-rearing villages in three districts of Kaabong and Karenga in northern Karamoja and Amudat in the southern fringes of the region bordering with the Republic of Kenya [Fig. 1]. Kaabong, Karenga and Amudat districts are comprised of three (3) rural counties/municipalities which in total contain 93 cattle-rearing parishes. There are an estimated 947,000 head of cattle in these three districts; the majority [*n* = 604,000] of which are in Kaabong and Karenga. There are an estimated 1,507 cattle in each of the 628 villages of Amudat, Kaabong and Karenga district [8].

### Sample size determination

Cluster sampling [9, 10] implemented in C Survey version 2.0 was used to calculate the number of villages needed to satisfy the set precision. The list of Amudat, Kaabong and Karenga villages (clusters) herein after called the sampling frame was obtained from the national bureau of statistics online database https://data.humdata.org/dataset/uganda-administrative-boundaries-admin-1-admin-3. This list was verified for completeness at the respective district lands and planning offices and corresponding Geographic Information System [GIS] coordinates. Data points for each of the selected villages were taken at the time of taking cattle blood samples. To minimize sampling errors resulting from the inherent variability between different samples of a larger population (a list of the latter being termed the sampling frame), villages were selected by simple random sampling.

Thirty-eight (38) clusters (55 cattle sampled in each) were required to estimate the prevalence of CBPP with this set precision. Randomly sampling 55 cattle in each of the 38 randomly selected clusters [Table 1] achieves the specified parameters for sample estimation. The number of animals per cluster is the mean of the number of animals per village [*n* = 1,507] in each of the selected 93 study parishes. The 38 clusters selected fulfilled the sampling assumption that the cluster means are normally distributed. In total, *n* = 2,313 cattle were sampled.

**Table 1:** Prevalence of Mmm antibodies in cattle sera from Karamoja region

District	*n* Sampled [% of N]	*n* Positive [%]	95% CI
Amudat	397 [17.3]	49 [12.3]	9.3–15.9
Kaabong	1,436 [62.4]	441 [30.7]	28.3–33.2
Karenga	467 [20.3]	95 [20.3]	16.8–24.3
Total [N]	2,300 [100]	585 [25.4]	23.7–27.3

### Individual animal sampling procedure

Every 30th animal in each village was sampled until the required number of at least 55 [in most cases 60 cattle] cattle per village [cluster]was sampled. This assumed an average of 1,507 cattle per study village. A temporary paint stick was used as a means of ensuring that only the required number of cattle were sampled in each village, and that each animal was sampled once.

### Blood sample collection and serum extraction

Selected cattle were physically restrained using ropes / crushes and blood collected from the jugular vein into serum separator vaccutainer tubes [SST]. To determine the seroprevalence of Mmm in cattle in Karamoja region, blood samples collected from cattle [*n* = 2,300] were screened for Mmm antibodies during the months of April and May 2021.Serum separator vacutainer tubes [SST] contain a clot activator gel that allows rapid blood clotting and facilitates fast serum separation. Three serum aliquots [1.5 mL each] were drawn from the SST vacutainer using a Pasteur pipette into pre-labelled cryogenic tubes 24 h after blood collection. These were packed into cryoboxes and temporarily stored at -20 °C at the district laboratory before transporting them back to the molecular biology laboratory at the college of veterinary medicine, animal resources and biosecurity, Makerere University once a week for a period of two months.

### Immunodetection of CBPP

Detection of Mmm IgG antibodies in cattle sera was done using commercially available IDEXX CBPP IgG tests [One IDEXX Drive, Westbrook, Maine 04092, USA] following Manufacturer’s guidelines. All kit reagents were first allowed to reach room temperature before they were used in the assay. The samples to be analyzed were first prepared on an uncoated (template) plate (sterile v-bottom culture plates) as follows: 100 µl of dilution buffer N.24 was dispensed into each well of the template plate. An extra 110 µl of dilution buffer N.24 was dispensed into two appropriate wells “CC” (i.e., CC = A1 and A2) such that CC has a total volume of 210 µl. This was followed by dispensing 11 µl of each undiluted strong positive control “SPC”, undiluted negative control “NC” into two appropriate wells (e.g., SPC = B1 and B2, NC = C1 and C2). 11 µl of undiluted samples was dispensed into the remaining wells of the template plate. The contents of the plate were gently homogenized using a multi-channel pipette before transferring 100 µl of this mixture into corresponding wells of the test microplate (provided in the kit). The microplate was covered with a plastic lid and incubated for 1 h at (37ºC ± 3ºC) after which the contents were discarded, and the plate washed with 300 µl of freshly prepared wash solution. This was followed by addition of 100 µl of freshly prepared conjugate and the plate similarly incubated for 30 min. Thereafter, the plate was similarly washed before adding 100 µl of substrate solution N.9 in each well and incubated for 20 min at (37ºC ± 3ºC) in the dark. 100 µl of stop solution was then added to each well and the plate optical density [OD] read at 450 nm in a BioTek 800 TS ELISA reader [5301, Stevens Creek Blvd, Santa Clara, CA 95,051 United States]. The average OD in the wells containing only the monoclonal antibody “MabC” [CC = wells A1 and A2] and wells containing the negative control were calculated. The percentage inhibition (SPI) was calculated as: (*SPI*) = 100*(*ODMabC* − *ODsample*)/(*ODMabC* − *ODNegative*)

The samples presenting SPI% less than or equal to 50% were considered positive whereas those presenting percentage inhibition greater than or equal to 50% were considered as negative for anti-CBPP antibodies.

### Data analysis

Descriptive statistics [prevalence, Odds ratios and their 95% confidence intervals-CIs] were computed in R-4.1.2 for Microsoft Windows using *anova*, *lme4, ggplot2, car, modx, sjPlot*, *Hmisc* and *epiR* packages. We fit mixed-effect binomial logistic regression models with random intercepts for villages using a step-wise-step up procedure with the *glmer* function in *lme4* package to explain the most important risk factors of seroprevalence of Mmm in cattle. Minimum adequate models performed better than intercept-only base line models in explaining the risk factors of cattle seroconversion to Mmm. Risk factors which significantly explained (using estimates for Analysis of Variance [ANOVA], Akaike Information Criteria [AIC] and Bayesian Information Criteria [BIC] for inclusion and exclusion) the variation within Mmm seroconversion were included in the final minimal adequate models. The effect variation [χ^2^, *p* = 0.05] of each risk factor was evaluated. The goodness of fit was tested using R^2^, C and Somers’ D values; with estimates closer to 1 considered as the best fit models. The ORs and their CIs for the intercepts and risk factors were determined at the 95% CI. All statistical analyses were performed in R statistical software version 4.0.1. ArcGIS v 10.8 (spatial analyst extension) was used to map prevalence estimates in different villages.

## Results

A quarter [25%] of all cattle sampled from the three Karamoja region districts were sero-positive for Mmm. Cattle sera from Amudat and Kaabong districts had the lowest [12.3%] and highest [30.7%] seroprevalence of MmmSC respectively [Table 1].

There was wide variation both at subcounty [3.3–87.1%] and at village [3.3–91.7%] levels in the seroprevalence of Mmm in cattle from Karamoja herds [Tables 2 and 3] with the highest and lowest seroprevalence detected in cattle that were roaming around Kaabong trading center and Kakamar Subcounty respectively [Table 2]. The majority [28/39] of the village herds had low seroprevalence levels [**≤** 30%] to Mmm [Table 3]. It is noted that at herd level, all villages in Karamoja region were seropositive to Mmm.

**Table 2:** Seroprevalence of Mmm in cattle [*n* = 2,300] in the respective Sub-counties in Karamoja region

Subcounty	District	*n* sampled (% of 2,300]	*n* Positive [%]	95% CI
Kaabong East	Kaabong	240[10.43]	57[23.8]	18.5–29.7
Kaabong T/C	Kaabong	116[5.04]	101[87.1]	79.6–92.6
Kaabong West	Kaabong	60[2.61]	19[31.7]	20.3–44.9
Kakamar	Kaabong	60[2.61]	4[6.7]	1.9–16.2
Kalapata	Kaabong	300[13.04]	57[19.0]	14.7–23.9
Kapedo	Karenga	60[2.61]	2[3.3]	0.4–11.5
Karenga	Karenga	60[2.61]	23[38.3]	26.1–51.8
Karita	Amudat	292[12.7]	37[13.0]	9.4–17.4
Kathile	Kaabong	120[5.22]	43[35.8]	27.3–45.1
Kathile South	Kaabong	120[5.22]	26 [21.7]	14.7–30.1
Kawakol	Karenga	120[5.22]	13[10.8]	5.9–17.8
Lobalangit	Karenga	120[5.22]	19[15.8]	9.8–23.6
Lodiko	Kaabong	60[2.61]	47[78.3]	65.8–87.9
Loleria	Kaabong	120[5.22]	24[20.0]	13.3–28.3
Loreria	Kaabong	60[2.61]	4[6.7]	1.9–16.2
Loroo	Amudat	105[4.57]	11[10.5]	5.4–17.9
Lotim	Kaabong	60[2.61]	43[71.7]	58.6–82.6
Loyoro	Kaabong	60[2.61]	9[15.0]	7.1–26.6
Sangar	Karenga	107[4.65]	38[35.5]	26.5–45.4
Sidok	Kaabong	60[2.61]	7[11.7]	4.8–22.6

**Table 3:** Seroprevalence of Mmm in cattle [*n* = 2,300] from selected villages in Karamoja region

Village	District	*n* sampled [% of 2,300]	*n* positive [%]	95% CI
Abiliyep	Amudat	49[2.13]	7[14.3]	5.9–27.2
Camp Swahili North	Kaabong	56[2.43]	46[82.1]	69.6–91.1
Cheptekol	Amudat	60[2.61]	14[23.3]	13.4–36.0
Cherelakoghum	Amudat	52[2.26]	6[11.5]	4.4–23.4
Iwakai	Amudat	60[2.61]	4[6.7]	1.9–16.2
Kopoth	Kaabong	60[2.61]	7[11.7]	4.8–22.6
Kachukul	Karenga	47[2.04]	17[36.2]	22.7–51.5
Kailob	Kaabong	60[2.61]	6[10.0]	3.8–20.5
Kajir	Kaabong	60[2.61]	47[78.3]	65.8–87.9
Kakira	Karenga	60[2.61]	9[15.0]	7.1–26.6
Kakochil	Karenga	60[2.61]	6[10.0]	3.8–20.5
Kakuma	Kaabong	60[2.61]	4 [6.7]	1.9–16.2
Kalapata	Kaabong	60[2.61]	9[15.0]	7.1–26.6
Kalere	Kaabong	60[2.61]	7[11.7]	4.8–22.6
Kodikdik	Amudat	60[2.61]	9[15.0]	7.1–26.6
Komuria North	Kaabong	60[2.61]	55[91.7]	81.6–97.2
Koteen	Kaabong	60[2.61]	6[10.0]	3.8–20.5
Locheger East	Kaabong	60[2.61]	12[20.0]	10.8–32.3
Lodwar West	Kaabong	60[2.61]	24[40.0]	27.6–53.5
Lokariowora	Kaabong	60[2.61]	28[46.7]	33.7–60.0
Lokuse	Kaabong	60[2.61]	8[13.3]	5.9–24.6
Lominyit	Kaabong	60[2.61]	7[11.7]	4.8–22.6
Lopedot	Amudat	56[2.43]	4 [7.1]	1.9–17.3
Lorengechewora	Kaabong	60[2.61]	43[71.7]	58.6–82.6
Moru-Arengan	Karenga	60[2.61]	7[11.7]	4.8–22.6
Moruangisigiria	Kaabong	60[2.61]	15[25.0]	14.7–37.9
Moruangangorok	Amudat	60[2.61]	5[8.3]	2.8–18.4
Moruangita	Kaabong	60[2.61]	18[30.0]	18.9–43.2
Moruerau	Kaabong	60[2.61]	19[31.7]	20.3–44.9
Morulem Centre	Kaabong	60[2.61]	4[6.7]	1.9–16.2
Muruanaido	Kaabong	60[2.61]	26[43.3]	30.6–56.8
Nabeteleng	Karenga	60[2.61]	23[38.3]	26.1–51.8
Nadwaramukuny	Kaabong	60[2.61]	4[6.7]	1.9–16.2
Nakorichokei	Karenga	60[2.61]	2[3.3]	0.4–11.5
Nameri	Kaabong	60[2.61]	15[25.0]	14.7–37.9
Naoyaro	Karenga	60[2.61]	10[16.7]	8.3–28.5
Naoyase	Karenga	60[2.61]	21[35.0]	23.1–48.4
Nariamaoi North	Kaabong	60[2.61]	20[33.3]	21.7–46.7
Simalok	Kaabong	60[2.61]	11[18.3]	9.5–30.4

A mixed-effect binomial logistic regression model with random intercepts for villages was fit to the Mmm seroprevalence data in a step-wise-step up procedure [marginal R2 = 0.147, conditional R2 0.319]. The final minimum adequate model performed significantly better than an intercept-only base line model [χ2 = 43.74, *p* = 8.32e^− 8^) and a good but not optimal fit model (C = 0.799, Somers’ D: 0.599). Generally, the odds of seroprevalence of Mmm increased [1.54–2.15] with increasing age [Table 4]. The final minimal adequate model indicated that location of cattle herds [districts] [χ2 = 18.92, *p* = 7.78e^− 5^] and overnight stay of cattle within Kraals significantly increased seroprevalence of Mmm among cattle in the different villages [χ2 = 14.58, *p* = 0.0001].

**Table 4:** Risk factors of seroconversion to Mmm by cattle [*n* = 2,300] from Karamoja region

Risk factors	*n*; analysed, [% of 2,300]	*n* positive [%]	OR	95% CI	*P*-value
Intercept			0.07	0.03–0.16	< 0.001
**Age [Months]**					
3–12	460[20.00]	79[3.43]	Ref	Ref	Ref
13–24	462[20.09]	112[4.87]	1.54	1.06–2.24	0.022
25–36	275[11.96]	81[3.52]	2.01	1.32–3.06	0.001
> 36	1103[47.96]	313[13.61]	2.15	1.55–2.98	< 0.001
**Location [District]**					
Amudat	397[17.26]	49[2.13]	Ref	Ref	Ref
Kaabong	1436[62.43]	441[19.17]	26.73	7.04–101.48	< 0.001
Karenga	467[20.30]	95[4.13]	19.81	4.18–93.95	< 0.001
**Overnight Kraal**					
No	573[24.91]	178[7.74]	Ref	Ref	Ref
Yes	1,727[75.09]	407[17.70]	0.08	0.03–0.27	< 0.001

**Fig. 2:**
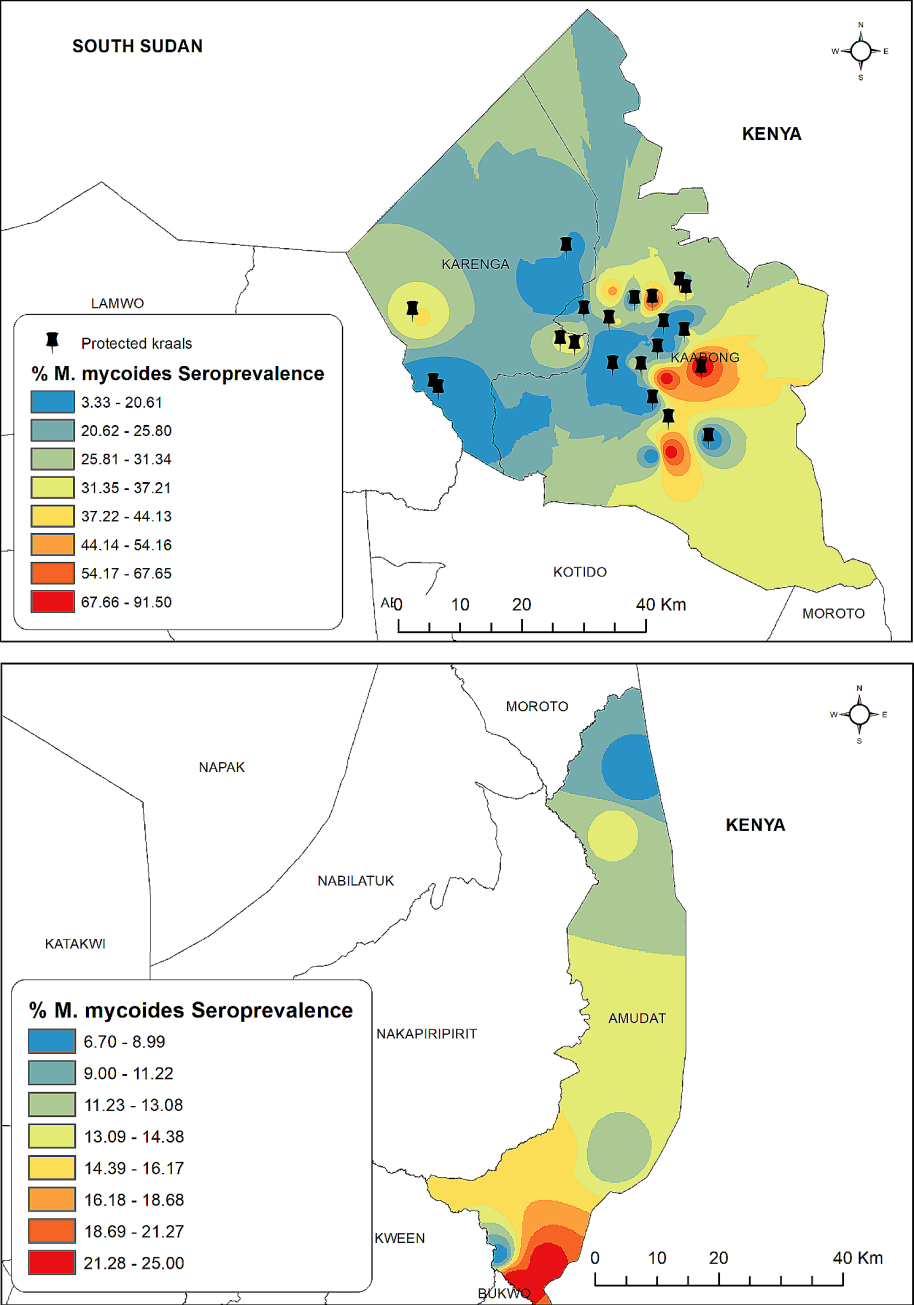
Spatial distribution of Mmm antibodies in cattle sera from Karamoja region. The figure was generated by the authors in ArcMap 10.7 software using open-source shape files. https://data.humdata.org/dataset/uganda-administrative-boundaries-admin-1-admin-3)

Highest seroprevalence of Mmm [> 31%] was recorded in cattle herds closest to the Sudan [Karenga District] and Kenya [Kaabong and Amudat Districts] international Boarders [Fig. 2].

## Discussion

This study provides update on status of Mmm exposure in the Karamoja region part of the triangle of North-eastern Uganda, North western Uganda and South-Eastern Sudan which has been for years a source of infection and spread of CBPP to the East African region and beyond. A quarter [25.4%; 95% CI: 23.7–27.3] of all cattle sampled in the three Karamoja region districts were sero-positive for Mmm. The prevalence of Mmm antibodies in this study is higher than what was reported in a previous study that involved screening of suspect outbreak herds; implying that Mmm might still be endemic in the Karamoja region [3]. The last vaccination against CBPP conducted in Karamoja region was in 2018 [11]. Whereas the Mmm-specific serological response following vaccination tends to be short-lived [12], IgG levels detected by cELISA following natural infection persist for about 52–56 weeks [13]. Thus it is highly likely that the seroprevalence herein is from natural exposure. For a livestock disease whose infection rate is about 90% and the mortality rate approaches 50%, an Mmm seroprevalence of 25% in apparently healthy animals possibly suggests that a large proportion of the infected could have died leaving behind 25% recovered animals, and or recovered and no longer sero-positive but are perhaps carrier lungers [14]. Much as the IDEXX CBPP competitive ELISA used in this study does not differentiate between recovered natural infection and vaccinated animals, the fact that antibodies following vaccination wane within 3 months period suggests that this sero-conversion could be from natural infection.

Generally, there was a spatial clustering of Mmm antibodies at village, subcounty and district levels. Cattle sera from Amudat and Kaabong districts had the lowest [12.3%] and highest [30.7%] seroprevalence of Mmm respectively [Table 1]. This observed spatial clustering of Mmm antibodies indicates that areas of high prevalence are involved in management practices that promote inter-herd comingling which promotes infection with Mmm. The seroconversion rate of 25% [either from recent infection or vaccination] indicates that 75% of all cattle in the sampled regions are naïve and prone to future infections. To prevent future CBPP outbreaks, there is need for government of Uganda and development partners to implement a risk-based CBPP vaccination program for Karamoja and other endemic regions of the country.

**Fig. 3:**
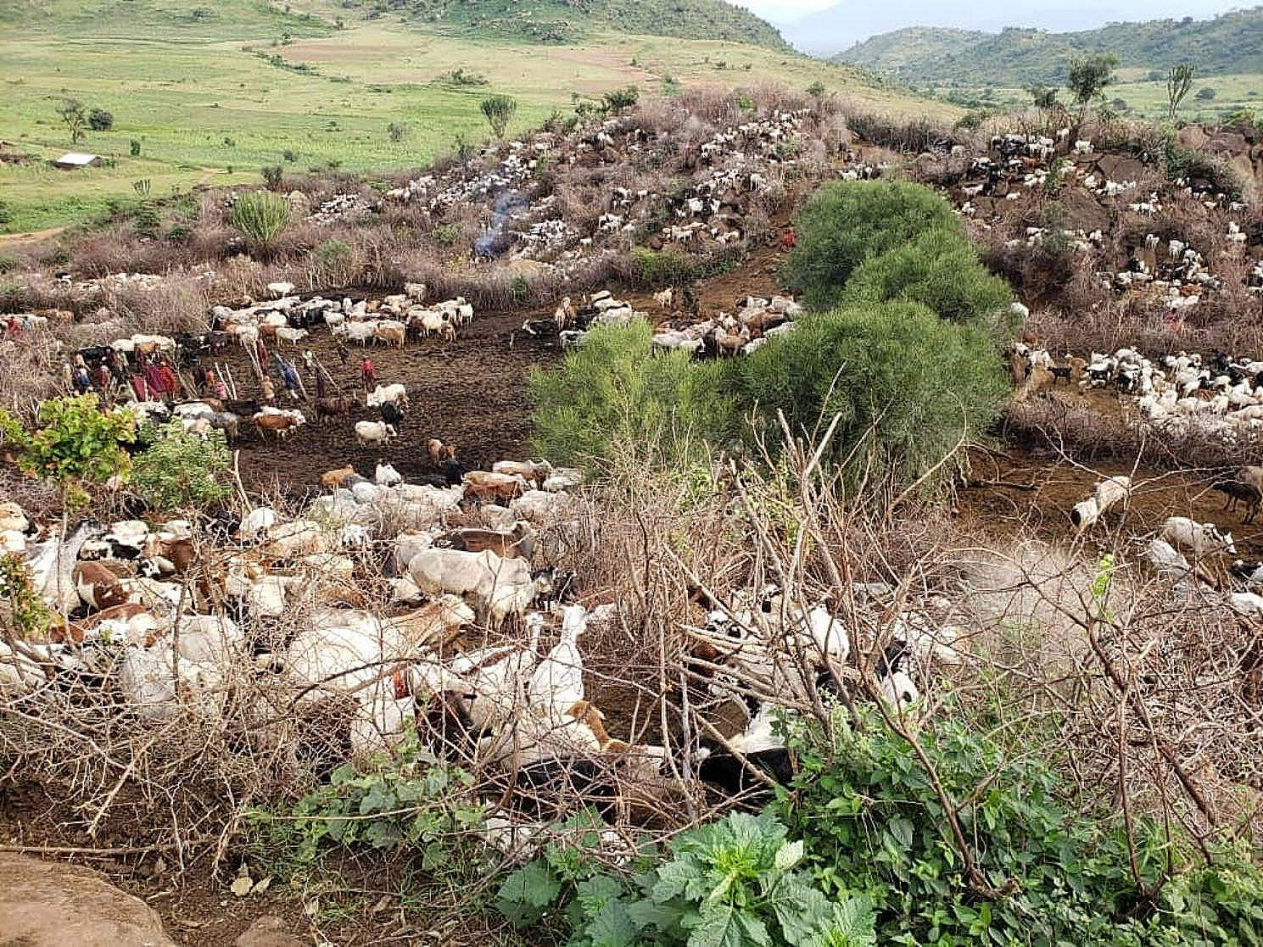
Protected cattle kraal; Kaabong District [Photo credit: Muhanguzi Dennis]

Significant risk factors of seroconversion with Mmm included: overnight stay of cattle within Kraals [χ2 = 14.58, *p* = 0.0001], increasing age [χ2 = 23.73, *p* = 2.85e^− 5^] and location of cattle herds in favour of herds closest to the Sudan [Karenga District] and Kenya [Kaabong and Amudat Districts] international Borders. In most parts of Karamoja region, animals from each parish [10–20 herds; *n* ∼ 5,000 cattle, ∼ 3,000 sheep and goats] are kept in large protected [by armed forces] Kraals [Fig. 3] overnight in order to keep them safe from cattle rustlers. This practice promotes comingling of such herds and hence transmissions of contagious diseases like CBPP between herds.

The observed spatial clustering of Mmm antibodies in herds along international borders agrees with published literature [15–18], that porous borders promote bidirectional movement of animal herds leading to transboundary animal diseases transmission and disease-endemic states. It is for this reason that herds closest to the Sudan [Karenga District] and Kenya [Kaabong and Amudat Districts] borders were most likely to be positive for Mmm antibodies. From these observations, CBPP vaccination programs in Karamoja region should consider adopting themselves to; targeting areas with high seroprevalence with preferential vaccination of animals that co-mingle in large mixed herds. Kraal members should consider making CBPP vaccination mandatory.

## Conclusion

In this study, slightly a quarter of all screened cattle [25.4%; 95% CI: 23.7–27.3] were seropositive to Mmm indicating that 75% of all cattle in the sampled regions are naïve and prone to future infections in this CBPP endemic region. Furthermore, increasing age, overnight stay in cattle kraals and location [certain districts, villages, herds and sub counties] of the cattle herds were the most significant risk factors of seroconversion with Mmm. These factors promote animal comingling and therefore Mmm transmission. It is therefore necessary that the government of Uganda and development partners implement a risk based CBPP vaccination program for Karamoja and other endemic regions of the country by targeting animals along the international borders and in hotspot villages/sub counties to prevent spread of Mmm to non-hotspot villages, sub counties and districts to prevent future CBPP incursions.

## Data Availability

The raw data that were analysed to generate results presented and discussed in this manuscript are available from the corresponding authors on reasonable request.

## Abbreviations

ANOVA: Analysis of Variance
CBPP: Contagious Bovine Pleuropneumonia
Mmm: *Mycoplasma mycoides* subspecies *mycoides*
MAAIF: Ministry of Agriculture Animal Industry and Fisheries
NADDEC: National Animal Disease Diagnostics and Epidemiology Centre
ELISA: Enzyme Linked Immunosorbent Assay
DIVA: Differentiate vaccinated from infected animals
FMD: Foot and Mouth Disease
GIS: Geographic Information System
SST: Separator vacutainer tubes
COVAB: College of Veterinary Medicine Animal Resources and Biosecurity
AIC: Akaike Information Criteria
BIC: Bayesian Information Criteria
